# Identification of glucocorticoid receptor in *Drosophila melanogaster*

**DOI:** 10.1186/s12866-020-01848-x

**Published:** 2020-06-15

**Authors:** Gloria Bartolo, Leandra O. Gonzalez, Saleem Alameh, C. Alexander Valencia, Mikhail Martchenko Shilman

**Affiliations:** 1 Henry E. Riggs School of Applied Life Sciences, Keck Graduate Institute, Claremont, CA 91711 USA; 2 Aperiomics, Inc., Sterling, VA 20166 USA; 3 Lake Erie College of Osteopathic Medicine, 1858 W Grandview Blvd, Erie, PA 16509 USA; 4 Department of Geriatrics, West China Hospital, Sichuan University, Chengdu, Sichuan China

**Keywords:** Glucocorticoid receptor, *Drosophila melanogaster*, Fruit fly, Estrogen receptor, Cortisol, Cortisone, *Culex quinquefasciatus*, Mosquitoes, *Saccharomyces cerevisiae*, Infection

## Abstract

**Background:**

Vertebrate glucocorticoid receptor (GR) is an evolutionary-conserved cortisol-regulated nuclear receptor that controls key metabolic and developmental pathways. Upon binding to cortisol, GR acts as an immunosuppressive transcription factor. *Drosophila melanogaster*, a model organism to study innate immunity, can also be immunosuppressed by glucocorticoids. However, while the genome of fruit fly harbors 18 nuclear receptor genes, the functional homolog of vertebrate GR has not been identified.

**Results:**

In this study, we demonstrated that while *D. melanogaster* is susceptible to *Saccharomyces cerevisiae* oral infection, the oral exposure to cortisol analogs, cortisone acetate or estrogen, increases fly sensitivity to yeast challenge. To understand the mechanism of this steroid-induced immunosuppression, we identified the closest genetic GR homolog as *D. melanogaster* Estrogen Related Receptor (*ERR*) gene. We discovered that *Drosophila* ERR is necessary for cortisone acetate- and estrogen-mediated increase in sensitivity to fungal infection: while ERR mutant flies are as sensitive to the fungal challenge as the wildtype flies, the yeast-sensitivity of ERR mutants is not increased by these steroids. Interestingly, the fungal cortisone analog, ergosterol, did not increase the susceptibility of *Drosophila* to yeast infection. The immunosuppressive effect of steroids on the sensitivity of flies to fungi is evolutionary conserved in insects, as we show that estrogen significantly increases the yeast-sensitivity of *Culex quinquefasciatus* mosquitoes, whose genome contains a close ortholog of the fly *ERR* gene.

**Conclusions:**

This study identifies a *D. melanogaster* gene that structurally resembles vertebrate GR and is functionally necessary for the steroid-mediated immunosuppression to fungal infections.

## Background

**Fig. 1:**
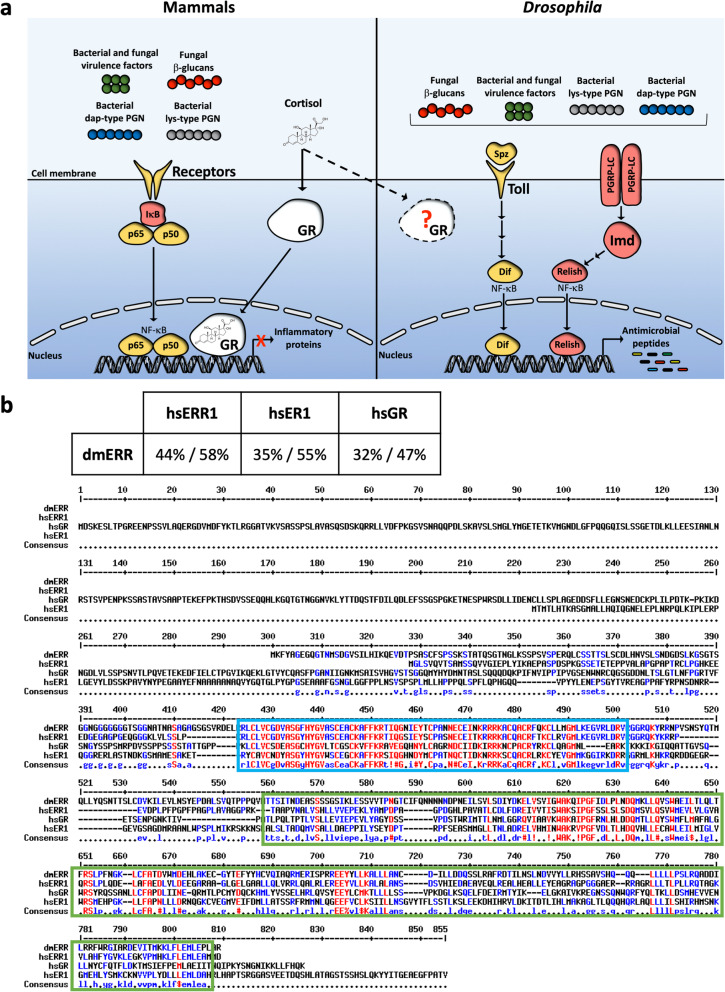
Identification of GR sequence-homolog in *Drosophila melanogaster*. **a** An illustration of the hypothesis for the existence of GR ortholog in fruit flies. During mammalian microbial infection (left panel), cell surface receptors, such as Toll-like receptors, detect bacterial and fungal cells. Once bound to microbial cells, mammalian receptors activate the expression of pro-inflammatory genes through the actions of Nuclear Factor κB (NF-κB). Steroids, such as cortisol, binds to a nuclear receptor, glucocorticoid receptor (GR), and trigger its translocation into the nucleus, where it represses NF-κB and the expression of pro-inflammatory genes. Similar pathways exist in insects (right panel), where two pathways detect invading microbial pathogens, and ultimately trigger the expression of antimicrobial peptides. Toll and Imd pathways both ultimately activate NF-κB transcription factors, which induce the transcription of antimicrobial peptides. We hypothesize the existence of a GR ortholog in fruit flies, capable of immunosuppressing infected insects in response to steroids. **b** Multiple amino acid sequence alignment between *D. melanogaster* Estrogen Related Receptor (dmERR), *Homo sapiens* ERR (hsERR1), *H. sapiens* estrogen receptor (hsER1), and *H. sapiens* glucocorticoid receptor (hsGR). The sequences of dmERR (Accession NP_648183), hsERR1 (Accession XP_016872802), hsER1 (Accession XP_016865870), and hsGR (Accession CAJ65924) were aligned using MultAlin software. The DNA binding domain and ligand binding domain are highlighted in blue and green boxes, respectively. Identical amino acids are shown in red. The extent (%) of the identity/similarity between *Drosophila* and human sequences is shown above the alignment

Glucocorticoids (GCs), steroid hormones produced in the adrenal cortex of the kidney [1], are important for regulating numerous physiological functions such as glucose metabolism and immune response [2]. Naturally occurring GCs in the human body are inactive precursor cortisone and its active metabolite, cortisol [1, 3, 4]. Cortisol, converted from cortisone via type 1 11ß-hydroxysteroid enzyme, functions by binding directly to the ligand binding domain (LBD) of glucocorticoid receptors (GRs) found within the cytosol of the target cell [1, 3, 4]. Once bound to cortisol, GR translocates from the cytosol to the nucleus where it homodimerizes [3, 4]. The cortisol-bound GR homodimer can act as a transcriptional activator of genes encoding anti-inflammatory proteins by allowing its DNA binding domain (DBD) to bind to glucocorticoid-responsive elements (GREs) [3, 4]. Concurrently, the cortisol-GR homodimer can bind to and inhibit the function of transcription factor Nuclear Factor κB (NF-κB), ultimately repressing the synthesis of NF-κB-dependent inflammatory proteins [3, 4] (Fig. 1a, left panel).

NF-κB is evolutionary-conserved and an integral part of the fruit fly *Drosophila melanogaster* innate immune response when challenged with entomopathogenic microbes. Upon the detection of microbial pathogens, *Drosophila* systemic humoral response activates two NF-κB-activating pathways, Imd and Toll, leading to the production of anti-microbial peptides (AMPs) [5, 6]. The Imd pathway is primarily induced by gram-negative bacteria by recognizing DAP-type peptidoglycan via PGRP-LC receptors located on the cell surface of enterocytes or fat body cells [5, 7]. Once the presence of bacteria is detected, the NF-κB transcription factor Relish activates the transcription of AMPs such as *diptericin.* A parallel cascade is triggered by the detection of gram-positive bacteria and fungi. The Toll pathway detects lysine-type peptidoglycan of gram-positive bacteria via PGRP-SA and GNBP1, as well as ß-glucans on the cell walls of fungi via GNBP3. This triggers the activation of the Toll receptor, and ultimately leads to the nuclear translocation of the NF-κB transcription factor Dif, activating the transcription of AMPs such as *drosomycin* [5] (Fig. 1a, right panel)*.* Recent studies show that sensing of the type of the bacterial cell wall is less stringent than previously thought and that both fly pathways are capable of detecting lys- and dap-peptidoglycan based on the accessibility of bacterial cell wall [7].

Human GR is a member of a nuclear receptor (NR) class of proteins. NRs are a superfamily of ligand regulated transcription factors activated by steroid hormones and various other lipid-soluble signals responsible for regulating a variety of processes such as embryonic development and metabolism [8–10]. Additionally, NRs are evolutionary conserved and represented in all animal phyla, including humans and *Drosophila* [11]. Human NR superfamily includes 48 NRs, which could be divided into six subfamilies based on their sequence similarity. Although *Drosophila* has only 18 NRs, they represent all 6 sub-families found in humans [8, 12, 13]. A previous study demonstrated that orally administered corticosteroid increases the sensitivity of *Drosophila* to pathogenic fungus, *Rhizopus oryzae* [14], alluding to the existence of unidentified fly GR (Fig. 1a). In this study, we report the identification and function of the *D. melanogaster* GR ortholog by examining yeast infection susceptibility upon steroid treatments.

## Results

### Genetic in silico search for GR homolog in the *Drosophila melanogaster* genome

Mammalian NR members of the same group share at least 80% identity in DBDs and at least 40% identity in LBDs [10]. Human GR belongs to the NR subfamily 3 (also known as steroid NRs), which is comprised of a total of nine family members, such as *Homo sapiens* estrogen-related receptor and estrogen receptor (hsERR1 and hsER1, respectively). Interestingly, *Drosophila* harbors only one member in the subfamily 3, which was called *D. melanogaster* ERR (dmERR) for its sequence homology to hsERR1 [8, 10, 15, 16]. We aligned the sequence of dmERR with sequences of several members of human NR subfamily 3 (hsERR1, hsER1, and GR) and observed an overall amino acid homology between 47 to 58% (Fig. 1b), with high similarity in the DBD (70–96%) and the LBD (55–57%) (Additional file 1). Due to a comparable degree of sequence homology of dmERR to GR and to hsERR1 (Fig. 1b), as well as the ability of corticosteroids to immunosuppress flies and humans to fungi [14], we hypothesized that dmERR may function as a functional ortholog of GR.

### Susceptibility of *Drosophila melanogaster* to *Saccharomyces cerevisiae* oral infection

Microbial pathogens, both bacterial and fungal, can infect *Drosophila* via different potential routes. A common route of access to microbial pathogens is the penetration of the gut, as *Drosophila* is naturally exposed to pathogens when foraging for food sources [17]. In addition to exposure to pathogens, *Drosophila* can also be exposed to non-pathogenic microbes such as *Saccharomyces cerevisiae*, a budding yeast that co-habitats with *Drosophila* in nature [18, 19]. Since the previous study demonstrated that a medically used GC decreases the survival of *Drosophila* to a human fungal pathogen [14], we set out to test whether naturally occurring GCs sensitize flies to non-pathogenic yeast, *Saccharomyces cerevisiae*.

**Fig. 2:**
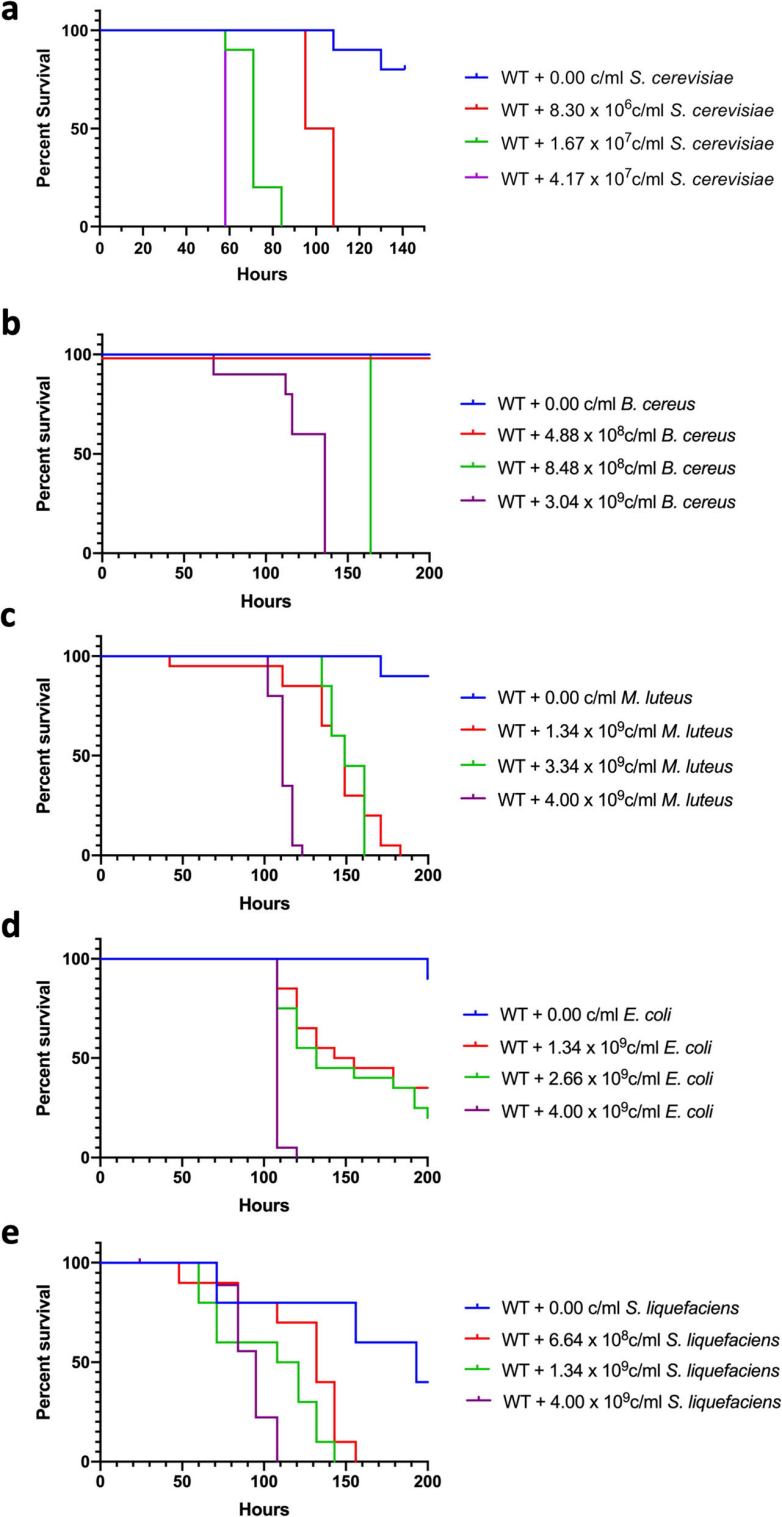
Sensitivity of female wildtype flies to microbial infections. Female Oregon-R wild type (WT) flies were orally challenged with different amounts of (**a**) *S. cerevisiae*, (**b**) *Bacillus cereus*, (**c**) *Micrococcus luteus*, (**d**) *Escherichia coli*, and (**e**) *Serratia liquefaciens*. Flies were fed in vials with 50 mM sucrose solution containing various microbial concentrations. Each condition contains ten flies. Vials are incubated at 30 °C and checked a minimum of twice per day for fly survival

Before the administration of GCs, we first assessed the sensitivity of *Drosophila* to *Saccharomyces* via oral exposure. Wildtype Oregon-R *Drosophila* flies were treated with sucrose solutions containing various concentrations of *S. cerevisiae* (Fig. 2a and Additional file 2), and compared to the pathogenicity of known entomopathogenic Gram-positive *Bacillus cereus* and *Micrococcus luteus*, as well as Gram-negative *Escherichia coli* and *Serratia liquefaciens* bacteria [7, 20–22] (Figs. 2b-e, respectively). Surprisingly, despite being known as natural symbionts [18, 19], we observed that *S. cerevisiae* is pathogenic to flies of both genders via continuous oral exposure. At 4.17 × 10^7^ yeast cells/ml in sucrose solution, fly median survival time (i.e. the time at which 50% of flies are dead) occurs after 58 h of exposure in both female and male flies (Fig. 2a). At 1.67 × 10^7^ yeast cells/ml, *S. cerevisiae* yields a median survival by 71 h in female flies (Fig. 2a) and 95 h in male flies (Additional file 2). A further half-fold decrease in fungal concentration (8.30 × 10^6^ cells/ml) leads to even longer median survival times: 101.5 h in female flies and 141 h in male flies (Fig. 2a and Additional file 2, respectively). Moreover, this data demonstrated that *S. cerevisiae* was more pathogenic to flies than any of the tested bacteria, since it took three orders of magnitude less of yeast cells to achieve a comparable death rate of flies caused by bacterial pathogens. Specifically, the fly death rate caused by 8.30 × 10^6^ yeast cells/ml (Fig. 2a) is comparable to the fly death rate caused by 4.00 × 10^9^ bacterial cells/ml (Figs. 2b-e).

While uninfected flies feeding solely on sucrose can survive for 10–14 days, some of the flies die at earlier times, such as after 100–150 h. Therefore, to study the effects of GCs on the sensitivity of *Drosophila* to yeast, a mid-concentration of 1.67 × 10^7^ fungal cells/ml in the sucrose solution was used for the remainder of the study, because it caused fly death well before 100 h.

### Increased susceptibility of *Drosophila* to fungal infection with oral exposure to cortisone acetate

**Fig. 3:**
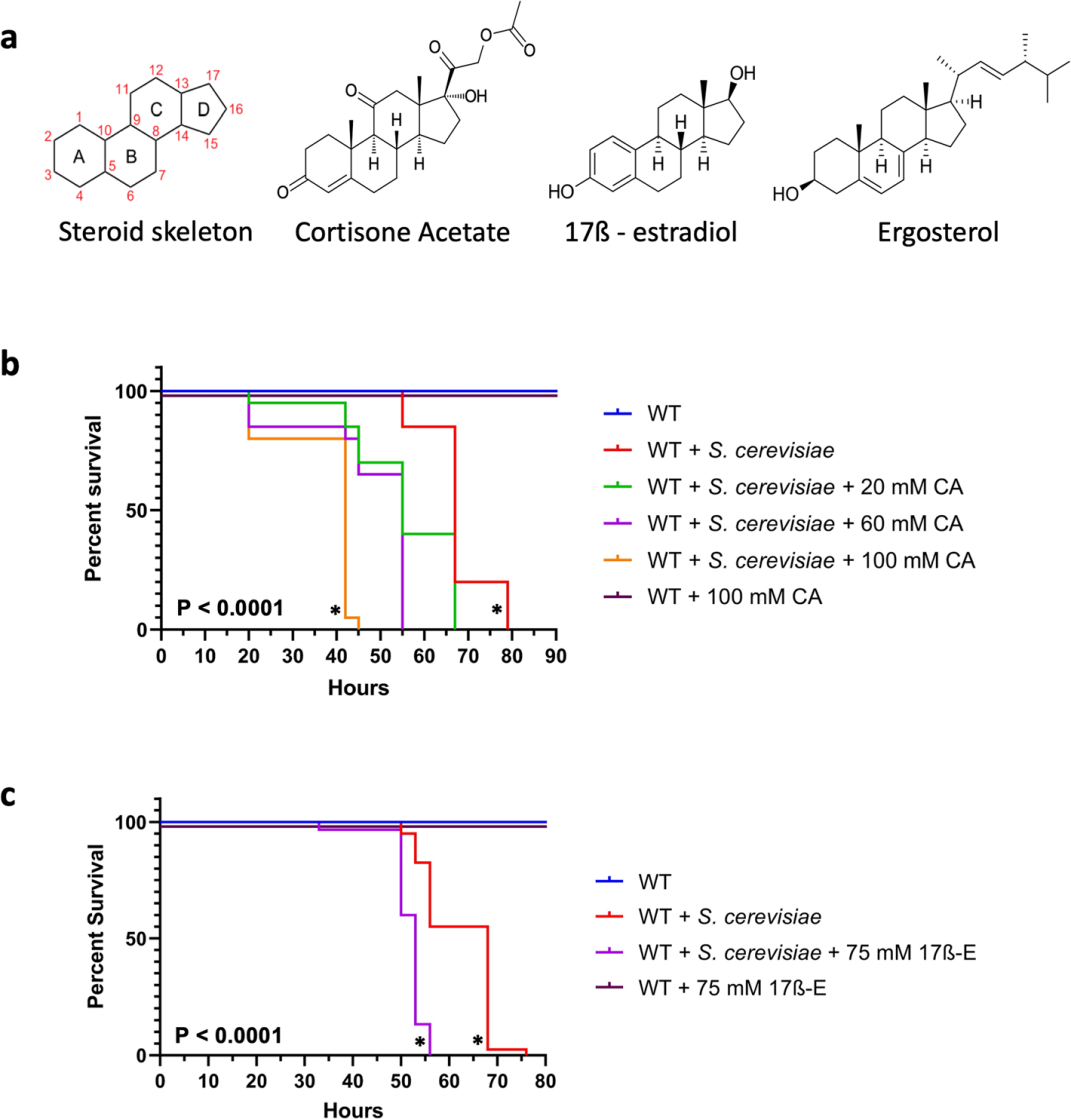
Steroid molecules increase the sensitivity of female wildtype flies to *Saccharomyces cerevisiae*. **a** Structures of steroid skeleton and cortisol analogs tested in this study: cortisone acetate, 17ß-estradiol, and ergosterol. (**b**-**c**) Female wild type (WT) flies were orally challenged as in Fig. 1 with 1.67 × 10^7^*S. cerevisiae* cells/ml with and without various concentrations of cortisone acetate (CA) (**b**) and 17ß-estradiol (17ß-E) (**c**). Uninfected flies exposed to 100 mM cortisone acetate or 75 mM 17ß-estradiol were included to test the toxicity of these compounds. *P*-values in b-c indicate statistical significance compared to the yeast-only condition (asterisks) on the basis of the Log-rank (Mantel-Cox) test

GCs are commonly prescribed for the treatment of numerous inflammatory and autoimmune diseases, as well as used as immunosuppressants to reduce the likelihood of organ rejection in organ transplant recipients [1, 3]. Cortisone acetate (CA), a synthetic GC converted to the naturally occurring cortisol within the human body (Fig. 3a), reduces the possibility of organ rejection while simultaneously increasing the susceptibility of recipients to fungal infections [22]. The significance of human CA-induced immunosuppression to fungal pathogens motivated us to select yeast for our subsequent tests. We tested the ability of CA to elicit a similar effect in *Drosophila* when orally exposed during a *S. cerevisiae* infection. Chamilos, et al. demonstrated increased susceptibility of wildtype flies to *Rhizopus oryzae* zygomycotic systemic infection when flies are orally exposed to 20 mg/ml (50 mM) of the synthetic GC, dexamethasone [14].

For our study, we orally provided CA to wildtype flies in conjunction with exposure to *S. cerevisiae* in sucrose solution. Various concentrations of CA, ranging from 20 mM to 140 mM, were provided to determine a CA concentration where flies of both genders were more susceptible to *S. cerevisiae* infection (Fig. 3b and Additional file 3). Additionally, we determined that none of the tested CA concentrations were toxic to uninfected flies. The concentrations of CA found to significantly increase the susceptibility of female and male flies to infection were 100 mM and 140 mM, respectively. Both concentrations for each gender led to decreased time to death. In males, who required exposure to a higher concentration of CA than females to increase susceptibility to *S. cerevisiae*, the median survival was reached 20 h sooner with CA compared to the control without CA. Females exhibited a similar effect on median survival when exposed to 100 mM CA: median survival was shortened by 25 h. These results show that, like in mammalian species, cortisone acetate increased the susceptibility of flies to fungi.

### Increased susceptibility of flies to fungal infection with oral exposure to mammalian sex hormone

We sought to test whether other known steroid ligands of human NR subfamily 3 could also increase *Drosophila* susceptibility to *S. cerevisiae* infection. Because dmERR is homologous to both hsERR1 and hsER1, we tested the ability of a mammalian ER ligand, the estrogen steroid hormone, 17ß-estradiol (17ß-E) (Fig. 3a), to affect fly susceptibility to *S. cerevisiae* infection. 17ß-E, a molecule with both anti- and pro-inflammatory effects [23], exhibits a steroid skeleton structure very similar to CA.

Since 17ß-E is a female hormone in mammals, we utilized only female flies to determine the effect of the compound on the susceptibility of flies to *S. cerevisiae* infection. 17ß-E was orally supplied within the concentration range as that of CA. At 75 mM, 17ß-E was more effective than CA in increasing the susceptibility of female wildtype flies to *S. cerevisiae* infection, without causing toxicity to uninfected flies (Fig. 3c). The median survival of infected flies occurred 15 h sooner in the presence of 17ß-E than without this compound. This data demonstrates that multiple steroid hormones could increase the sensitivity of flies to fungal challenge.

### Effect of estrogen on *Culex quinquefasciatus* mosquitoes during fungal infection

**Fig. 4:**
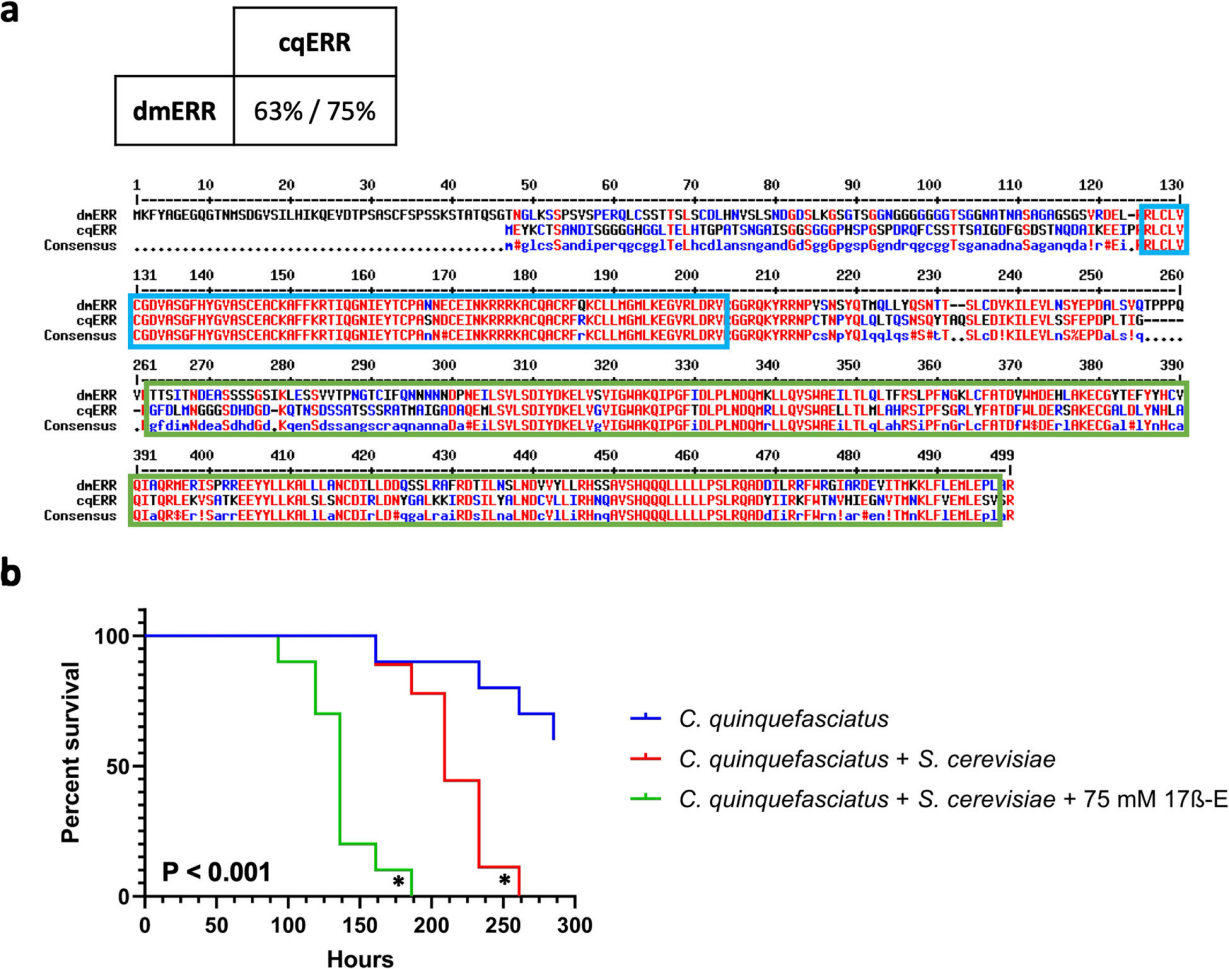
The effect of 17ß-estradiol on the yeast-sensitivity of *Culex quinquefasciatus* mosquitoes. **a** Multiple amino acid sequence alignment between *D. melanogaster* Estrogen Related Receptor (dmERR) and *C. quinquefasciatus* ERR (cqERR). The sequences of dmERR (Accession NP_648183) and cqERR (Accession EDS37237) were aligned using MultAlin software as in Fig. 1b. The DNA binding domain and ligand binding domain are highlighted in blue and green boxes, respectively. Identical amino acids are shown in red. The extent (%) of the identity/similarity between *Drosophila* and *Culex* sequences is shown above the alignment. **b** Female *C. quinquefasciatus* mosquitoes were orally challenged with 3.3 × 10^7^*S. cerevisiae* cells/ml with and without 75 mM of 17ß-estradiol (17ß-E). *P*-value indicates statistical significance compared to the yeast-only condition (asterisks) on the basis of the Log-rank (Mantel-Cox) test

To determine whether steroids affect the sensitivity of other insects to yeast infections, we searched for ERR homologs in other species. The genome of *Culex quinquefascuiatus* mosquitoes harbors a close homolog of fly *ERR*, with 63% amino acid identity and 75% similarity (Fig. 4a). The function of *C. quinquefascuiatus ERR* homolog, locus EDS37237, is currently unknown and annotated as *ERR* based on its sequence similarity to other *ERR* genes. Because mosquitoes contain an *ERR* ortholog, we tested the ability of a steroid molecule to affect the sensitivity of yeast-infected *C. quinquefascuiatus*, who just like *Drosophila,* is a member of the insect order Diptera [24].

Before the exposure to 17ß-E, we determined 3.3 × 10^7^ fungal cells/ml as the minimal lethal dose necessary to kill female adult *C. quinquefascuiatus* mosquitoes, aged 4–5 days. Female mosquitoes were then orally exposed to 17ß-E, the same concentration shown to immunosuppress flies (Fig. 4b). Like fly experiments, we observed that 75 mM of 17ß-E increased the sensitivity of mosquitoes to *S. cerevisiae* and decreased median survival time by 73 h. This data shows the steroid hormone-mediated increased sensitivity to fungal infection is conserved in multiple insect species harboring the *ERR* gene.

### *Drosophila* ERR is necessary for CA- and 17ß-E-mediated increased sensitivity to fungal infection

**Fig. 5:**
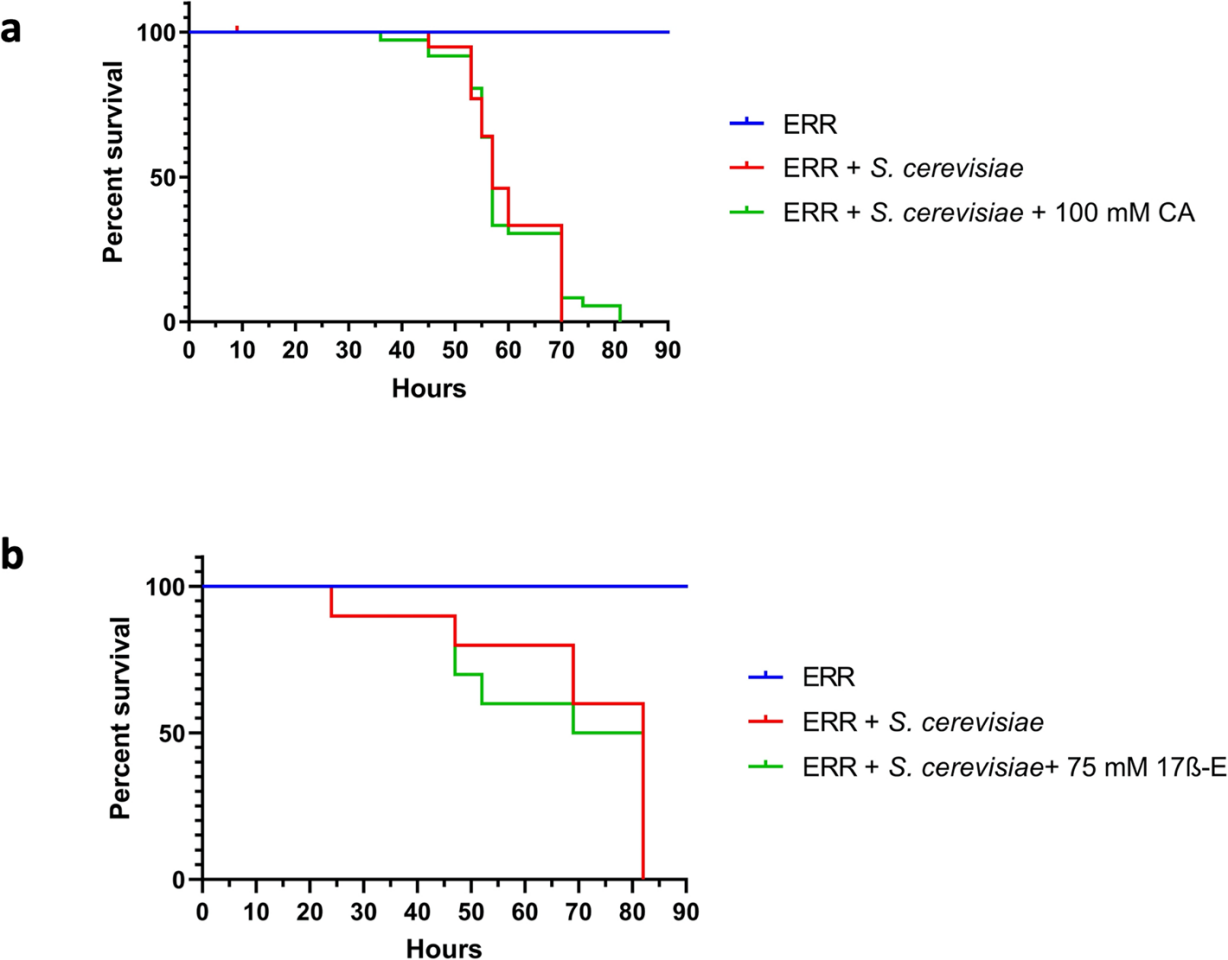
The yeast-sensitivity of ERR mutant flies is unaffected by steroid molecules. Female Estrogen Related Receptor (ERR) mutant flies were orally challenged as in Fig. 3 with 1.67 × 10^7^*S. cerevisiae* cells/ml with and without 100 mM cortisone acetate (**a**) and 75 mM 17ß-estradiol (**b**)

To determine whether dmERR acts as a functional human GR ortholog, female *ERR* homozygous loss-of-function mutant flies were challenged with *S. cerevisiae* in the presence or absence of CA (Fig. 5a) or 17ß-E (Fig. 5b). We observed that in the absence of steroids, *ERR* mutant flies exhibited similar sensitivity to yeast infection as wildtype *Drosophila* (median survival at 50–70 h) (Fig. 5), allowing for a parallel analysis between both fly strains. Interestingly, with the oral exposure of CA or 17ß-E, dmERR mutant flies did not show increased susceptibility to *S. cerevisiae* infection at concentrations found to increase the susceptibility of wildtype flies (100 mM for CA and 75 mM for 17ß-E). The median survival time of dmERR mutant flies exposed to *S. cerevisiae* + CA or *S. cerevisiae* + 17ß-E was comparable to the median survival time of wildtype flies to *S. cerevisiae* alone, which illustrated the inability of dmERR mutant flies to become more susceptible to a fungal infection in the presence of CA or 17ß-E. This data showed that *Drosophila* ERR is necessary for steroid-mediated increased sensitivity of flies to fungal infection.

### Determination of the effect of fungal ergosterol on the susceptibility of *Drosophila* to yeast infection

**Fig. 6:**
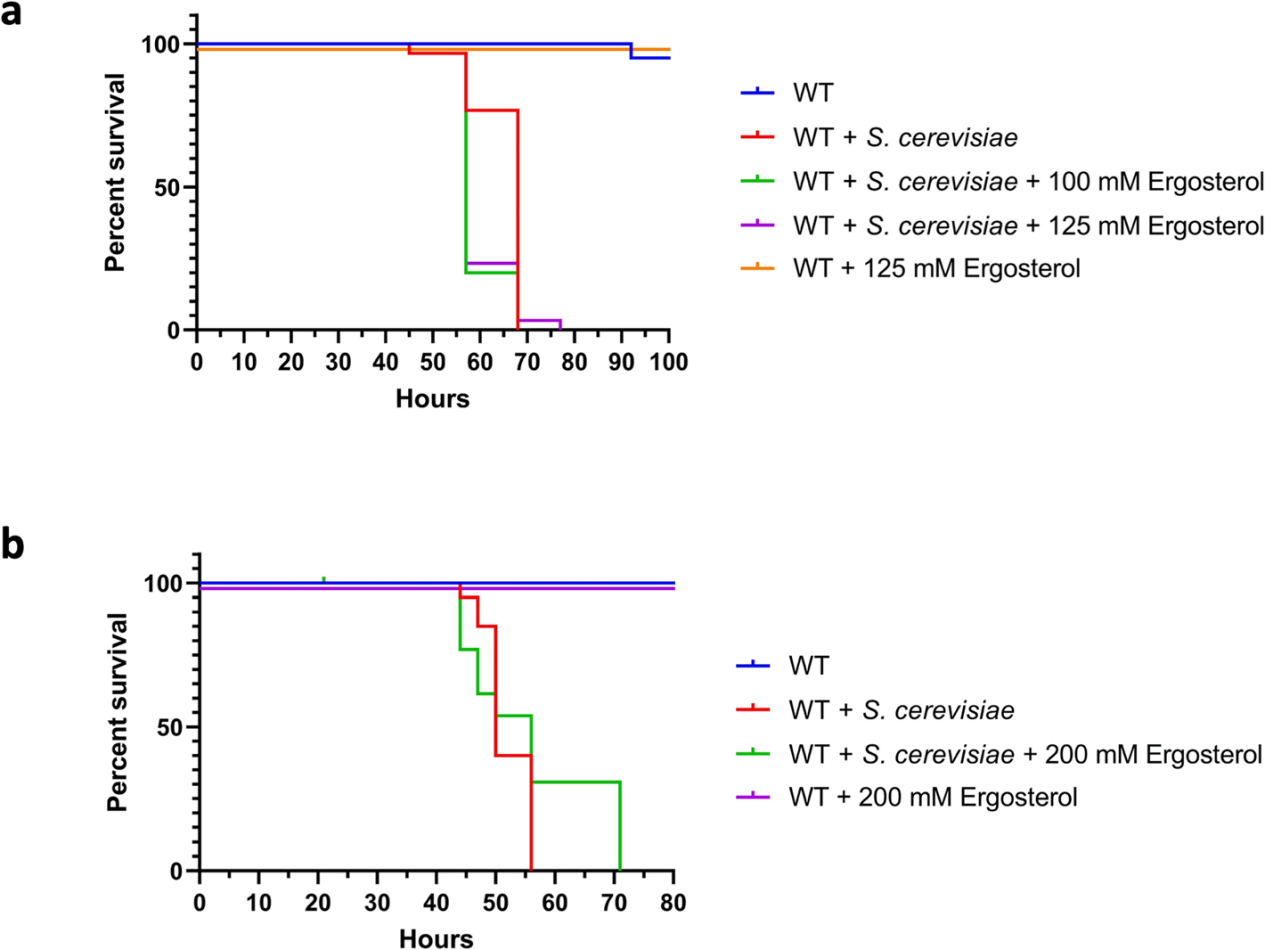
Fungal ergosterol does not affect the yeast-sensitivity of female wildtype flies. Female wild type (WT) flies were orally challenged as in Fig. 3 with 1.67 × 10^7^*S. cerevisiae* cells/ml with and without indicated concentrations of ergosterol

While CA and 17ß-E are naturally occurring soluble and plasma-circulating mammalian steroids [3, 25], we investigated the effect of fungal steroids on the sensitivity of flies to yeast. Previously, *S. cerevisiae* has been shown not to generate extracellular soluble steroids [26] and instead only produces cell membrane-bound steroid-like ergosterol, which is important for maintaining membrane fluidity, permeability, and structure [27]. Like CA and 17ß-E, ergosterol exhibits the steroid skeleton but with a longer side chain on the 17th carbon atom (Fig. 3a). We explored the hypothesis that after ingestion of *S. cerevisiae* cells, fungal ergosterol may affect the sensitivity of *Drosophila* to *S. cerevisiae*. Ergosterol was exogenously provided to female wildtype flies at a range from 100 to 200 mM (Fig. 6). At concentrations tested previously for CA and 17ß-E, ergosterol yielded no increased susceptibility of flies to *S. cerevisiae* infection (Fig. 6a). Even at double the concentration (200 mM), no effect on fly sensitivity was also seen (Fig. 6b).

## Discussion

Here, we presented dmERR is required for steroid-mediated increased fly sensitivity to fungal infections. Previous studies have reported roles for dmERR in carbohydrate metabolism and hypoxic responses [28–30]. Other research has focused on the possible role of dmERR in mitochondrial biogenesis [31], as hsERR1 has been shown to have a role in generating mitochondria [32]. In humans, the expression of medium-chain acyl coenzyme A dehydrogenase (MCAD), which is an enzyme that mediates the mitochondrial beta-oxidation of fat, is regulated by an ERR-α response element (ERRE) present in the 5′-flanking region. In COS-7 cells, hsERR1 interacts with the MCAD nuclear receptor response element 1 (NRRE-1). hsERR1 may regulate cellular energy balance by controlling the expression of MCAD through the NRRE1 [33]. Recent studies demonstrated that mitochondria are critical in stimulating innate immune signaling. Specifically, released mitochondrial DNA (mtDNA) and mitochondria-derived reactive oxygen species (mtROS) activate innate immune responses, such as inflammasome, sGAS-STING, and NF-κB signaling pathways [34]. In addition, changes in mitochondria and metabolic pathways (TCA cycle, oxidative phosphorylation, and fatty acid oxidation) induce transcription in immune cells. For example, M1 macrophages with an impaired TCA cycle have a pro-inflammatory response, and M2 macrophages perform β-oxidation to produce anti-inflammatory responses [35]. Additionally, mitochondria are known to induce the inflammatory response: mitochondrial antiviral signaling and NLRP3 can be activated by mitochondria [36]. Moreover, the mass and mobility of mitochondria are affected by fission and fusion that affect the immune functions [37]. In immune cells, mitochondria are located close to the endoplasmic reticulum (ER), which allows cells to control metabolism that is essential for immune functions [37]. Our results are the first to allude the role of dmERR in *Drosophila* immunity, specifically in increasing the susceptibility of flies to fungal infections when orally exposed to steroid molecules. Specifically, we have shown the ability of synthetic GC, CA, to increase the susceptibility of flies to fungal infection when orally presented. This is consistent with dmERR being the only homolog for members of the human NR subfamily 3, in which GR is included. dmERR is necessary for steroid-induced immunosuppression of flies and is highly homologous to GR. Thus, we propose that dmERR is the functional ortholog of GR.

Not all molecules exhibiting the steroid backbone elicit the same effect on fly sensitivity to fungal infection. Tested concentrations of ergosterol did not increase fly sensitivity to *S. cerevisiae* infection. Unlike CA and 17ß-E, which are transported to their target via the bloodstream in mammals, ergosterol is not soluble and/or mobile. Rather, it is integrated within the cell wall of *S. cerevisiae* and may not be readily bioavailable for fly ingestion. It is possible ergosterol elicits an effect at a higher concentration than tested, but such concentration may not be physiologically relevant. The inability of ergosterol to sensitize flies to *S. cerevisiae* could be either because ergosterol is not a ligand for dmERR or because the supplied ergosterol is soluble and out of the context of the fungal membrane.

Similarly to human ERR, the ligand for dmERR has yet to be discovered. Several studies have alluded to the existence of a ligand for dmERR [13, 38, 39], but a definitive ligand has not been reported. If dmERR is indeed the functional ortholog of human GR, there exists a greater possibility of the existence of a natural ligand for dmERR. If one is found to exist, *Drosophila* may be used as a model to identify a ligand for hsERR1 in humans, relieving the NR of its orphan status and providing a deeper understanding of the biological functions of dmERR and/or hsERR1. Additionally, *D. melanogaster*’s sophisticated innate immune system has largely evolved to combat bacterial and fungal pathogens relevant to the understanding of human inflammatory conditions [40]. In response to pathogenic challenges, AMPs are released through two primary pathways that involve evolutionarily conserved components, including Toll and Toll-like receptors, as well as NF-κB, tumor necrosis factor-α, and JAK/STAT signaling [41]. Thus, human mutations identified in hsERR1 can be studied by creating humanized dmERR to elucidate the innate immune effects of those genetic changes, as *Drosophila* lack adaptive immunity.

Fruit flies have thus far only one identified steroid hormone, 20-hydroxyecdysone (20E). Ecdysone was shown to regulate both immunity and major developmental transitions in the fly, such as metamorphosis [42]. Two *Drosophila* nuclear receptors are known to be receptors for 20E: ecdysone receptor (EcR) and ultraspiracle (USP). EcR binds to 20E, heterodimerizes with USP [43], and activates the expression of a large set of genes known to function in cell motility, cell shape, and phagocytosis [42]. Ecdysone-regulation in *Drosophila* was shown to be essential for hemocyte immune functions and survival after infection: 20E induces the phagocytosis in *Drosophila* hemocytes, and larvae lacking ecdysone-activated hemocytes are defective in bacterial phagocytosis and are susceptible to oral bacterial infections. In contrast, the results presented here show that the role of *Drosophila* ERR is to suppress the immunity of flies, which may be needed to counterbalance the positive effect of EcR on immunity.

While this study observes the necessity of fly ERR for the steroid-mediated immunosuppression, future studies will focus on the mechanism by which GC affects ERR, such as whether GC binds to ERR and induces its nuclear translocation, followed by its nuclear activity. Mammalian GRs are known to affect the immunity by two nuclear mechanisms: by binding directly to and repressing the transcriptional activity of NF-κB, as well as by binding directly to the promoters of immunity-related genes and regulating their transcription. Moreover, GRs affect immunity-related cytoplasmic proteins by a third non-nuclear mechanism via activating cytoplasmic phosphatidylinositol 3-kinase and protein kinase Akt, leading to the activation of secondary messengers nitric oxide [3]. Investigating whether fly ERR is affecting immunity-related genes and processes analogously to the three mammalian mechanisms will help us understand how GC influences ERR and innate immunity pathways.

## Conclusions

This study identifies a *D. melanogaster* gene that structurally resembles vertebrate GR and is functionally necessary for the steroid-mediated immunosuppression to fungal infections.

## Methods

### *Drosophila* rearing

*Drosophila melanogaster* strains were housed at 25 °C with 12-h light/dark cycles and fed on standard cornmeal-molasses-agar fly medium with yeast flakes. Wildtype experiments were conducted with Oregon-R, selected for their rapid egg-laying ability (Bloomington *Drosophila* Stock Center (BDSC) stock #2376), *Drosophila* aged 4–5 days. Experiments with homozygous *ERR* (BDSC stock #28467) *Drosophila* utilized unaged flies at the time of the experiments.

### *Drosophila* oral feeding survival assay

*Saccharomyces cerevisiae* diploid strain YEF473, ATCC® 200970 [44] was used as the infective agent for all *Drosophila* survival assays. *S. cerevisiae* was incubated on YPD medium at 30 °C. Overnight cultures were grown in YPD at 30 °C at 180 rpm for 14–16 h.

*Bacillus cereus* (ATCC 10987), *Escherichia coli* (C600), *Micrococcus luteus* (ATCC 4698), and *Serratia liquefaciens* (ATCC 27592) were used. *B. cereus* and *E. coli* were cultured in Lysogeny broth (LB) at 37 °C. *M. luteus* was grown in LB at 25 °C. *S. liquefaciens* was incubated in Tryptic Soy Broth (TSB) at 30 °C overnight.

Flies were infected according to the microbial intestinal infection methods described previously in Nehme, et al [45] with the following modifications. *Drosophila* vials were prepared by placing three 25 mm diameter circles of extra-thick Whatman blotting paper (Bio-Rad Laboratories, catalog #1703965) at the bottom of the vials and capping with a foam plug. *S. cerevisiae* overnight cultures were centrifuged, and the pellets resuspended in 50 mM sucrose solution to a final desired optical density (0.83 OD, 1.7 OD or 3.3 OD) at 600 nm (OD_600_). OD_600_ values were converted to cells/ml (OD_600_ of 1.0 corresponds to approximately 10^7^ cells/ml) [46]. Bacterial infections were carried out analogously, except at higher cells/ml (converted from OD_600_ values according to McFarland’s scale) [47, 48]. Depending on the experiment, steroid molecules were added to the fungal sucrose solution. 17ß-estradiol (catalog #10006315), cortisone acetate (catalog #23798), and ergosterol (catalog #19850) were all purchased from the Cayman Chemical Company. Each prepared *Drosophila* vial contained 2.5 ml of its respective solution, which was absorbed by the Whatman paper found at the bottom of the vial. Flies were anesthetized by CO_2_, separated by gender, and placed into the *Drosophila* vials, with ten flies in each vial. Vials were incubated at 30 °C and checked a minimum of twice per day for fly survival.

### Mosquito rearing

*C. quinquefasciatus* mosquitoes were obtained from a colony maintained by Benzon Research (Carlisle, PA, USA). Mosquitoes were reared and maintained at 28 °C and 80% relative humidity in 30 × 30 × 30-cm cages with 12-h light/dark cycles. Adult mosquitoes were maintained on 10% sucrose ad libitum, while larvae were fed a 1:1:1 mixture of bovine liver powder (Carlisle, PA, USA). For experiments, adult female mosquitoes aged 4–5 days were used.

### Mosquito oral infection survival assay

*S. cerevisiae* diploid strain YEF473, ATCC® 200,970 [44] was used as the infective agent for all *C. quinquefasciatus* survival assays.

*C. quinquefasciatus* were infected similar to the technique used to infect *Drosophila*, as described above, but include the following modifications. *C. quinquefasciatus* vials were prepared by placing 5 × 5-cm of extra-thick Whatman blotting paper (Bio-Rad Laboratories, catalog #1703965) in square-bottom, polypropylene *Drosophila* bottles and capped with a foam plug. *S. cerevisiae* overnight cultures were centrifuged, and the pellets resuspended in 50 mM sucrose solution to a final optical density at 600 nm (OD_600_) of 3.3 OD. OD_600_ values were converted to cells/ml (OD_600_ of 1.0 corresponds to approximately 10^7^ cells/ml) [46]. 17ß-estradiol (catalog #10006315) steroid molecules, purchased from Cayman Chemical Company, were added to the fungal sucrose solutions. Each prepared *C. quinquefasciatus* bottle contained 10 ml of its respective solution and absorbed by the Whatman paper found at the bottom of each vial. Mosquitoes were anesthetized using CO_2,_ separated by gender, and placed in the prepared vials, with ten mosquitoes in each vial. Vials were incubated at 30 °C and checked a minimum of once a day for mosquito survival.

## Supplementary information

**Additional file 1.** The amino acid sequence similarity between DNA and ligand binding domains of dmERR with hsERR1, hsER1, and hsGR. The amino acid sequences of DNA binding domain (DBD) and ligand binding domain (LBD) are compared between dmERR, hsERR1, hsER1, and hsGR. The extent (%) of the identity/similarity is shown for each domain.

**Additional file 2. **Sensitivity of male wildtype flies to *Saccharomyces cerevisiae* infection. Male Oregon-R wild type (WT) flies were orally challenged with different amounts of *S. cerevisiae*. Flies were fed in vials with 50 mM sucrose solution containing various yeast concentrations. Each condition contains ten flies. Vials are incubated at 30 °C and checked a minimum of twice per day for fly survival.

**Additional file 3. **Cortisone acetate increases the sensitivity of male wildtype flies to *Saccharomyces cerevisiae*. Male wild type (WT) flies were orally challenged as in Fig. 1 with 1.67 × 10^7^*S. cerevisiae* cells/ml with and without various concentrations of cortisone acetate (CA). Uninfected flies exposed to 140 mM cortisone acetate were included to test the toxicity of this compound. *P*-value indicates statistical significance compared to the yeast-only condition (asterisks) on the basis of the Log-rank (Mantel-Cox) test.

## Data Availability

All data generated or analyzed during this study are included in this published article [and its supplementary information files].

## Abbreviations

GC: Glucocorticoid
LBD: Ligand binding domain
GR: Glucocorticoid receptor
DBD: DNA binding domain
GRE: Glucocorticoid responsive elements
NF-κB: Nuclear factor κB
AMP: Anti-microbial peptide
NR: Nuclear Receptor
hsERR1: Human estrogen-related receptor 1
hsER1: Human estrogen receptor 1
dmERR: *Drosophila* estrogen-related receptor
CA: Cortisone acetate
17ß-E: 17ß-estradiol
