# Supplementary material for: Identification of glucocorticoid receptor in Drosophila melanogaster
Source: BMC Microbiol. 2020 Jun 15;20:161. doi: 10.1186/s12866-020-01848-x (PMC7296755; doi:10.1186/s12866-020-01848-x)
Supplement: Supplementary file 1 — Additional file 1. The amino acid sequence similarity between DNA and ligand binding domains of dmERR with hsERR1, hsER1, and hsGR. The amino acid sequences of DNA binding domain (DBD) and ligand binding domain (LBD) are compared between dmERR, hsERR1, hsER1, and hsGR. The extent (%) of the identity/similarity is shown for each domain. [file 12866_2020_1848_MOESM1_ESM.docx]

|  | | **hsERR1** | **hsER1** | **hsGR** |
| --- | --- | --- | --- | --- |
| **dmERR** | **DBD** | **89% / 96%** | **67% / 80%** | **57% / 70%** |
|  | **LBD** | **36% / 55%** | **30% / 55%** | **34% / 57%** |

**Additional file 1: The amino acid sequence similarity between DNA and ligand binding domains of dmERR with hsERR1, hsER1, and hsGR.**

The amino acid sequences of DNA binding domain (DBD) and ligand binding domain (LBD) are compared between dmERR, hsERR1, hsER1, and hsGR. The extent (%) of the identity/similarity is shown for each domain.
