# Supplementary material for: Identification of glucocorticoid receptor in Drosophila melanogaster
Source: BMC Microbiol. 2020 Jun 15;20:161. doi: 10.1186/s12866-020-01848-x (PMC7296755; doi:10.1186/s12866-020-01848-x)
Supplement: Supplementary file 2 — Additional file 2. Sensitivity of male wildtype flies to Saccharomyces cerevisiae infection. Male Oregon-R wild type (WT) flies were orally challenged with different amounts of S. cerevisiae. Flies were fed in vials with 50 mM sucrose solution containing various yeast concentrations. Each condition contains ten flies. Vials are incubated at 30 °C and checked a minimum of twice per day for fly survival. [file 12866_2020_1848_MOESM2_ESM.docx]

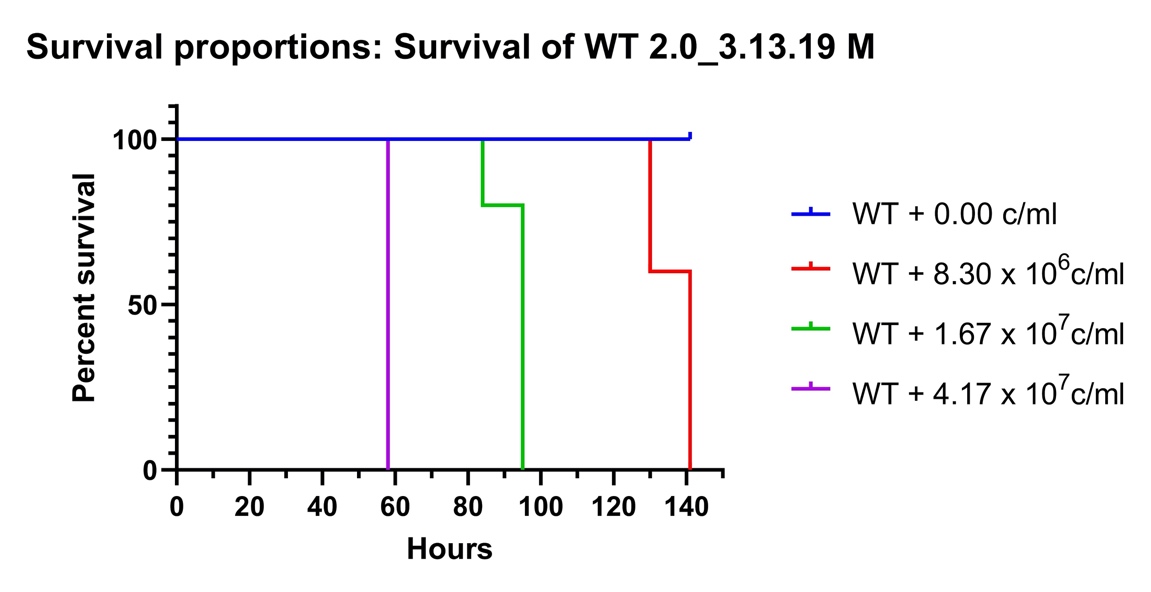


**Additional file 2: Sensitivity of male wildtype flies to *Saccharomyces cerevisiae* infection.**

Male Oregon-R wild type (WT) flies were orally challenged with different amounts of *S. cerevisiae*. Flies were fed in vials with 50 mM sucrose solution containing various yeast concentrations. Each condition contains ten flies. Vials are incubated at 30˚C and checked a minimum of twice per day for fly survival.
