# Supplementary material for: Identification of glucocorticoid receptor in Drosophila melanogaster
Source: BMC Microbiol. 2020 Jun 15;20:161. doi: 10.1186/s12866-020-01848-x (PMC7296755; doi:10.1186/s12866-020-01848-x)
Supplement: Supplementary file 3 — Additional file 3. Cortisone acetate increases the sensitivity of male wildtype flies to Saccharomyces cerevisiae. Male wild type (WT) flies were orally challenged as in Fig. 1 with 1.67 × 10^7^S. cerevisiae cells/ml with and without various concentrations of cortisone acetate (CA). Uninfected flies exposed to 140 mM cortisone acetate were included to test the toxicity of this compound. P-value indicates statistical significance compared to the yeast-only condition (asterisks) on the basis of the Log-rank (Mantel-Cox) test. [file 12866_2020_1848_MOESM3_ESM.docx]

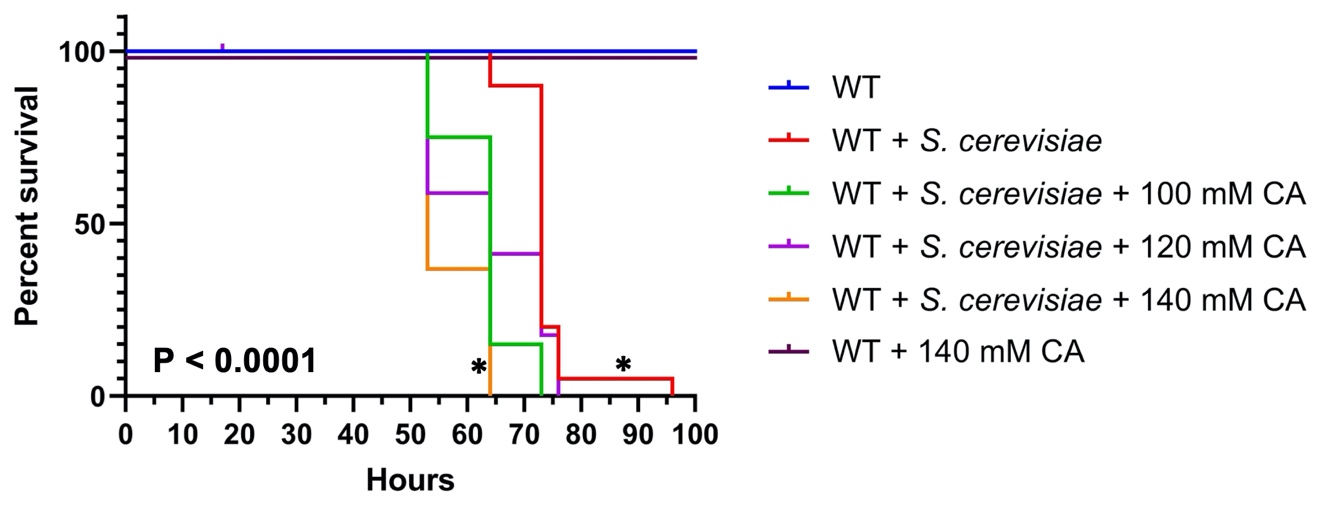


**Additional file 3: Cortisone acetate increases the sensitivity of male wildtype flies to *Saccharomyces cerevisiae*.**

Male wild type (WT) flies were orally challenged as in Fig. 1 with 1.67 x 10^7^ *S. cerevisiae* cells/ml with and without various concentrations of cortisone acetate (CA). Uninfected flies exposed to 140 mM cortisone acetate were included to test the toxicity of this compound. *P*-value indicates statistical significance compared to the yeast-only condition (asterisks) on the basis of the Log-rank (Mantel-Cox) test.
